# A computational model of the basal ganglia dual pathways organization

**DOI:** 10.1186/1471-2202-14-S1-P146

**Published:** 2013-07-08

**Authors:** Jean Liénard, Benoît Girard

**Affiliations:** 1 Department of Mathematics and Science Programs, Washington State University, Vancouver, WA 98686, USA; 2 Institut des Systèmes Intelligents et de Robotique, Pierre and Marie Curie University - Centre National de la Recherche Scientifique, UMR 7222, 75005 Paris, France

## 

We elaborated computational models of the whole primate basal ganglia (BG) with the aim of answering to the long standing questions [1,2] of the relationships between the external part of the globus pallidus (GPe) and its internal part (GPi). In particular, we aimed to solve the apparent contradiction between a) anatomical studies showing that the GPi is an important target of a gabaergic inhibition from GPe [3] that is further thought to be powerful, as the synaptic targets in GPi are close to the soma [4], and b) electrophysiological studies that record very similar activities in both nuclei, at rest [5], in directional arm reaching tasks [6] and during eye closures [7]. Furthermore, the BG are supposedly the substrates of a generic selection process among cortical inputs, but the classical explanation of this selection stemming from the segregation between a direct pathway (with the "direct" striatal neurons targeting straight the GPi) and an indirect pathway (with the "indirect" striatal neurons targeting the GPe) is not compatible with primate anatomical data showing a nearly total overlap in the efferences of the striatal neurons.

To address these problems, we parameterized mean-field models with the aim of fitting to an unprecedented large body of quantitative data obtained in primates. These data fall into two categories: a) anatomical data that directly constrain the parameter numerical value (they are the mean number of varicosities along the axons or the localization of the synapses along the dendrites) and b) electrophysiological data that can be used to constrain the model in simulation (they are the firing rate recorded either at rest, or when a given neurotransmitter is experimentally blocked by an antagonist). We built two matching fitness functions assessing how models were respecting these data, and we optimized them with the NSGA-2 multi-objective evolutionary algorithm [8].

We obtained more than one thousand different sets of parameters maximizing both fitnesses, and each of these models constitute a different answer to the initial paradox. By specifically examining the GPe -> GPi parameters, we found that a moderate strength of this projection is not antagonist with similar activities in both nuclei. Broader analysis led to the prediction that the GPe and GPi inputs are unbalanced, the former receiving stronger afferences from the striatum and the subthalamic nucleus than the latter.

We also tested the selection capability of our plausible models by mimicking a classical arm-reaching task performed in monkeys [6], and we show that (1) the plausible basal ganglia models that we obtained are performing selections, indicating that the mainstream theory of selection is still valid without the postulate of a segregation into direct and indirect pathways, and that (2) this selection is performed more efficiently with a diffused projection pattern from the GPe into the GPi, as compared to a focused projection.
